# The effect of hemicelluloses on biosynthesis, structure and mechanical performance of bacterial cellulose-hemicellulose hydrogels

**DOI:** 10.1038/s41598-024-72513-w

**Published:** 2024-09-17

**Authors:** Vadym Chibrikov, Piotr Mariusz Pieczywek, Justyna Cybulska, Artur Zdunek

**Affiliations:** Institute of Agrophysics, Polish Academy of Sciences, Doświadczalna 4 Str., 20–290 Lublin, Poland

**Keywords:** Bacterial cellulose, Hemicellulose, Hydrogels, Enzymolysis, Structure, Mechanical properties, Atomic force microscopy, Biomaterials, Composites

## Abstract

The primary plant cell wall (PCW) is a specialized structure composed predominantly of cellulose, hemicelluloses and pectin. While the role of cellulose and hemicelluloses in the formation of the PCW scaffold is undeniable, the mechanisms of how hemicelluloses determine the mechanical properties of PCW remain debatable. Thus, we produced bacterial cellulose–hemicellulose hydrogels as PCW analogues, incorporated with hemicelluloses. Next, we treated samples with hemicellulose degrading enzymes, and explored its structural and mechanical properties. As suggested, difference of hemicelluloses in structure and chemical composition resulted in a variety of the properties studied. By analyzing all the direct and indirect evidences we have found that glucomannan, xyloglucan and arabinoxylan increased the width of cellulose fibers both by hemicellulose surface deposition and fiber entrapment. Arabinoxylan increased stresses and moduli of the hydrogel by its reinforcing effect, while for xylan, increase in mechanical properties was determined by establishment of stiff cellulose–cellulose junctions. In contrast, increasing content of xyloglucan decreased stresses and moduli of hydrogel by its weak interactions with cellulose, while glucomannan altered cellulose network formation via surface deposition, decreasing its strength. The current results provide evidence for structure–dependent mechanisms of cellulose–hemicellulose interactions, suggesting the specific structural role of the latter.

## Introduction

The primary plant cell wall (PCW) is a highly specialized structure composed primarily of water and polysaccharides, while glycoproteins, phenols, minerals, and enzymes serve as minor components. Cellulose, hemicelluloses and pectins are the three main types of PCW polysaccharides with mass fractions of 20–40%, 20–30% and 30–50% of dry mass, respectively^1^. Cellulose is a linear polysaccharide of β-D-glucose linked by 1 → 4 glycosidic bonds, which is considered to be the major load-bearing component of PCW ^2^. Hemicelluloses consist predominantly of 1 → 4-linked monosaccharides of β-D-glucose, β-D-glucuronic acid, D-xylose, β-D-mannose, β-D-galactose, α-L-fucose, and α-L-arabinose^3^. Due to structural similarity to cellulose, hemicelluloses are thought to enable the formation of a broad hydrogen bonding network that determines the mechanical properties of PCW^4^. And for the last 60 years, it allowed the scientific community to evolve through the various concepts of primary plant cell wall structure^5–10^. The very first PCW models considered cellulose as an organized network, immersed in amorphous matrix of PCW components^5^. However, the following PCW models suggested there is an option PCW matrix polysaccharides (hemicellulose, pectin) to define intefiber interactions by covalent linkages^6,11^, direct fiber coating^7^, formation of interfiber tethers^8^, as well as forming polysaccharide-linking amalgam in close proximity to fiber surface^9,10^. To the best of our knowledge, all the PCW models reported have declared undeniable role of hemicelluloses in the integrity of the PCW scaffold. So do the modern theory, supporting the notion that cellulose is the main load-bearing component of PCW, while the key component of PCW mechanical properties—interfiber adhesion—is determined by the hemicellulose-mediated interactions.

The main insights into the structure and mechanical properties of PCW can be reached by the decomposition of PCW constituents in muro^12^, inhibition of its biosynthesis^10^, in vitro solubilisation^13^, or chemical modification^14^. Beside of that, fiber networks such as those of bacterial cellulose (BC) mimic PCW fiber networks on a macroscale, being homogeneous, repeatable, modifiable, and easy to handle. The homogeneous fiber distribution and its layered structure are the key factors for the stiffness of the BC fiber network, similar to those of PCW.

Despite the most common BC-hemicellulose fiber networks, such as BC-xyloglucan, BC-pectin, and BC-xyloglucan-pectin have already been studied^15–17^, there is still a lack of understanding on the role of other hemicelluloses and their content on structure and mechanical properties of cellulose-hemicellulose fiber networks. Moreover, previous explorations of mechanical properties of cellulose‑hemicellulose fiber networks were limited to their study in the dry state, while its description and exploration as a water‑swollen network, analogous to in vivo PCW, was barely revealed^18^.

This study aimed to investigate the effect of different hemicelluloses on the process of biosynthesis, composition, structure and mechanical properties of cellulose-hemicellulose hydrogels, that mimic PCW. The study was particularly focused on the effect of post-biosynthetic enzymolysis of hemicelluloses. Current approach aimed to define the structure-dependent interaction of hemicelluloses with cellulose and to evaluate its consequences for the structure and mechanical properties of cellulose-hemicellulose hydrogels. Since the hemicelluloses differ in chemical composition (molecular weight, monosaccharide composition), their incorporation in cellulose fiber network and enzymatic degradation affect the load bearing junctions in cellulose-hemicellulose hydrogels, so that affecting its mechanical properties. Because the hemicelluloses, used in the study, represent the most common in monocots and dicots, the results obtained can serve as a reference for a wide range of PCW, their models, as well as get across the possible commercial use of respected biomaterials. To our knowledge, this study is the only integrated analysis of the role of hemicelluloses and its enzymolysis on the mechanical properties of cellulose-hemicellulose hydrogels, combined with structural studies, and exploration of its chemical composition.

## Materials and methods

### Sample preparation

#### Biosynthesis of BC-hemicellulose hydrogels

Pure BC was produced in Hestrin-Schramm (HS) culturing medium with no hemicelluloses^19^. The culturing medium was autoclaved at 121 ºC for 11 min, and then brought to reach room temperature. Then, 100 mL of culturing medium was transferred to sterile 250 mL Erlenmeyer flasks followed by 2 mg of two‑day fresh colonies of *Komagataiebacter xylinum* ATCC‑53524 (LGC Standards, UK) bacteria strain. To obtain flat sheets, BC hydrogels were produced statically at 30 ± 1 ºC for 10 days. After production, samples were stirred with deionized water for 4 days to remove debris from culturing medium, bacteria trapped, proteins, as well as loosely attached hemicelluloses. BC-hemicellulose hydrogels were stored in a 0.02% mass sodium azide solution at 4 ± 1 ºC before further use^20^. The dry yield of biosynthesis of raw samples was calculated in triplicate as a dry mass of BC synthesized per 1 L of culturing medium.

In contrast, BC‑hemicellulose hydrogels were prepared in a HS medium with an addition of respected hemicelluloses^17^. The medium was prepared by dissolving 2% mass glucose (Sigma-Aldrich, USA), 0.5% mass casein peptone (Sigma-Aldrich, USA), 0.5% mass yeast extract (Thermo Fisher Scientific, USA), 0.27% mass anhydrous disodium phosphate (Chempur, Poland), and 0.115% mass citric acid monohydrate (Chempur, Poland) in deionized water. The pH of the culturing medium was then adjusted to 5.0 with 1 M NaOH/HCl. One of the four hemicelluloses—tamarind xyloglucan (P-XYGLN, > 95% purity, Megazyme, Bray, Ireland), beechwood xylan (P-XYLNBE-10G, > 95% purity, Megazyme, Bray, Ireland), medium-viscosity wheat flour arabinoxylan (P-WAXYL, > 95% purity, Megazyme, Bray, Ireland), and low-viscosity konjac glucomannan (P-GLCML, > 98% purity, Megazyme, Bray, Ireland) was added to culturing media. Monosaccharide composition and the weight average molecular weight of hemicelluloses used in current study is provided in Table 1.^21^.

**Table 1:** Monosaccharide composition and weight average molecular weight of hemicelluloses, used in current study.

Hemicellulose	Monosaccharide composition (% dry polysaccharide mass)							Weight average molecular weight (kDa)
	*Araf*	*Gal*	*Glc*	*GlcA*	*Man*	*Xyl*	Other	
Xylan				11.3		86.1	2.6	158.3
Arabinoxylan	37.8					61.7	0.5	323.0
Xyloglucan	2.0	17.0	45.0			34.0	2.0	802.5
Glucomannan			40.0		60.0			950.0*

Abbreviation of *Araf* stands for arabinose, *Gal* galactose, *Glc* glucose, *GlcA* glucuronic acid, *Man* mannose, *Xyl* xylose. Data on monosaccharide composition and weight average molecular weight was provided by a manufacturer. Asterisk sign * refers to data from^22^.

The mass fractions of hemicelluloses in the culturing medium of respected hydrogels are presented in Table 2.

**Table 2:** Samples studied, code names, and mass fractions of hemicelluloses in culturing medium.

Type of hemicellulose	Mass fraction in culturing medium (% w/v)			
	0.00	0.25	0.50	1.00
Arabinoxylan	BC	AX0.25	AX0.50	AX1.00
Glucomannan		KGM0.25	KGM0.50	KGM1.00
Xyloglucan		XGY0.25	XGY0.50	XGY1.00
Xylan		XYL0.25	XYL0.50	XYL1.00

*BC* stands for pure bacterial cellulose, other abbreviations stand for the specific hemicelluloses used.

**Fig. 1:**
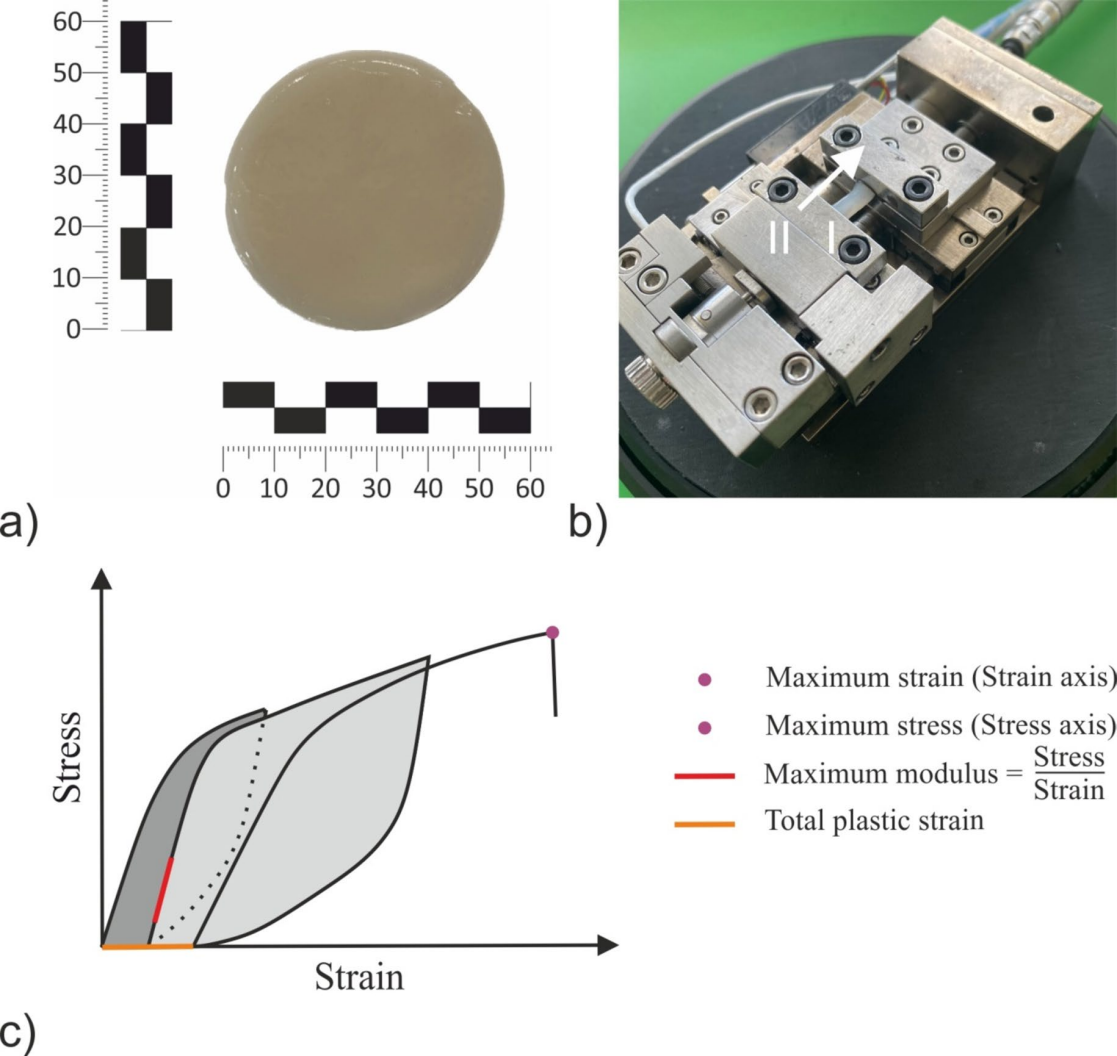
Uniaxial tensile test with cyclic load of BC hemicellulose hydrogels: (**a**) BC hemicellulose hydrogel on a millimeter precision scale; (**b**) tensile stage microtester with marked: (I) strip of BC hemicellulose hydrogel, (II) direction of loading; (**c**) schematic three cycle stress strain curve. The colored lines, points and areas define mechanical properties evaluated, while the annotation on the right provides the definition.

At the beginning of the preparation of BC-hemicellulose hydrogels, an increasing turbidity of the culturing medium was observed, corresponding to the initiation of BC biosynthesis^23^, followed by the formation of a hydrogel pellicle on medium-air interphase. After terminating of cultivation and washing, BC‑hemicellulose hydrogels appeared as a round pellicles with an approximate diameter of 55 ± 2 mm, and highly hydrated (∼98% mass of water; Fig. 1a). In the following sections, current samples were defined as *raw*.

#### Alkali treatment and enzymolysis

Alkali treatment and enzymolysis were performed to ensure depolymerization and removal of hemicelluloses of BC-hemicellulose hydrogels. Initially, samples were washed with deionized water to remove excess sodium azide. Then, samples were treated with 0.1 M NaOH at 80 ± 1 ºC for 30 min in triplicate, to remove proteins. Subsequently, the samples were washed in deionized water at 100 ± 1 ºC for 60 min, which is supposed not to affect hemicellulose composition, and not to change the structure and properties of BC^15,24^. Commercial enzymes (all—Megazyme, Ireland), specific to hemicelluloses used in current study, were chosen for enzymolyses. Enzyme solutions were prepared according to the manufacturer's recommendations, and the following data are presented in Table 3.

**Table 3:** Hemicelluloses, hemicellulose-specific enzymes used, the composition of the enzyme solutions, and the reactions catalysed by enzymes.

Hemicellulose	Enzyme	Solution composition	Reaction catalyzed
Arabinoxylan	Endo-β-1 → 4-xylanase (EC 3.2.1.8)	0.1 M sodium phosphate buffer (pH = 6), 0.5 mg/mL BSA	Enzymolysis of xylan to oligosaccharides
	α-L-arabinofuranosidase (EC 3.2.1.55)	0.1 M sodium acetate buffer (pH = 4)	Enzymolysis of α-1 → 2- and α-1 → 3-linked L-arabinofuranose sidechains of arabinoxylan
Glucomannan	Endo-1 → 4-β-mannanase (EC 3.2.1.78)	0.1 M sodium acetate buffer (pH = 4)	Enzymolysis of β-1 → 4-D-mannosidic linkages of glucomannan
Xyloglucan	Xyloglucan-specific endo-β-1 → 4-glucanase (EC 3.2.1.151)	0.1 M sodium acetate buffer (pH = 5.5), 1 mg/mL BSA	Enzymolysis of β-1 → 4-D-glycosidic linkages of xyloglucan
Xylan	Endo-β-1 → 4-xylanase (EC 3.2.1.8)	0.1 M sodium phosphate buffer (pH = 6), 0.5 mg/mL BSA	Enzymolysis of xylan to oligosaccharides

*EC* enzyme commission number, *BSA* bovine serum albumin.

Enzymatic treatment of BC-hemicellulose hydrogels was conducted by immersing hydrogels in 10 U/mL enzyme solutions up to $$6.67\times {10}^{-3} \frac{g of hydrogel dry mass}{mL of enzyme solution}$$ concentration. Thus, approximately 5 g of BC-hemicellulose hydrogels were immersed in 15 mL of enzyme solutions with an enzyme activity of 10 U/mL, where 1 U stands for conversion of 1 µmol of substrate per minute. The enzyme solutions were left diffusing within the samples for 3 h at 21 ± 1 ºC, followed by 5 h at 40 ± 1 ºC^25^. In the case of BC-arabinoxylan hydrogels, two successive enzymolyses were performed—first with endo-β-1 → 4-xylanase, followed by α-L-arabinofuranosidase. Pure BC was treated with 0.1 M sodium phosphate buffer (pH = 6) under the same conditions. Each treatment was terminated by washing hydrogels first in respected buffers, followed by deionized water. Finally, hydrogels were stored in a 0.02% mass sodium azide solution at 4 ºC prior to further use. In the following sections, the samples were defined as *treated*.

### Monosaccharide composition

Monosaccharide composition of both raw and treated BC-hemicellulose hydrogels was conducted according to^26^ with slight modifications. Oven-dried and milled samples with a mass of appr. 0.1 g were first incubated at 80 ºC for 72 h in 2 M hydrochloric acid in methanol (Sigma-Aldrich, USA), followed by hydrolysis with 2 mL of 3 M trifluoroacetic acid (Merck, Germany) at 100 ºC for 7 h. Hydrolyzed samples were incubated at 70 ºC for 1 h in a mixture of 1 mL of high-performance liquid chromatography (HPLC) water (Chempur, Poland), 0.05 mL of 0.3 M aqueous sodium hydroxide and 0.05 mL of 0.5 M 1-phenyl-3-methyl-5-pyrazolone (Thermo Scientific Chemicals, USA) solution in methanol. After that, samples were neutralized with 0.05 mL of 0.3 M aqueous hydrochloric acid (Chempur, Poland), and sample monosaccharides were extracted threefold with chloroform (Merck, Germany). Reference samples of monosaccharides and uronic acids (arabinose, galactose, galacturonic acid, glucose, glucuronic acid, mannose, rhamnose, and xylose; all—Sigma-Aldrich, Germany) were processed the same way.

Samples were analysed using a HPLC system of S1130 pump, S5300 sample injector, S4120 column oven, and S3350 photodiode array detector at 246 nm wavelength (all—Sykam GmbH, Germany). For HPLC analysis, the ZORBAX Eclipse XDB analytical column, and Eclipse XDB–C18 guard column (all—Agilent Technologies, USA) were used. For each sample, monosaccharide analysis was performed in triplicate.

### Surface topography

Surface topography imaging was performed with an atomic force microscope (MultiMode 8-HR, Bruker, USA) in ScanAsyst in Air™ tapping mode on samples oven-dried at 45 ± 1 ºC up to constant mass. Two randomly chosen pieces of an approximate area of 15 mm^2^ were cut out and stuck to atomic force microscope (AFM) metal specimen disks. A SCANASYST-AIR AFM cantilever (Bruker, USA) was used for imaging. Overall captured area for single image was set to 2 $\times$ 2 µm with an image resolution of 1.95 nm per pixel. Images were processed with *Gwyddion 2.48* software^27^ for correction of data artefacts. For each sample, 10 images were captured to obtain a representative number of data. Surface topography imaging of samples was performed at a temperature of 19 ± 1 ºC, and a relative humidity of 33 ± 2% Then, the average width was evaluated for 100 random cellulose fibers. In the current paper, the term *microfiber* characterizes a structural unit of cellulose chains of identical dimensional configuration, gathered in crystalline/semi-crystalline/amorphous domains. The term *fiber* refers to a group of microfibers, interacting with each other by weak forces, which structurally appear as a single unit, and in terms of applied load provide a similar response. For 10 random surface topography images of each type of bacterial cellulose-hemicellulose hydrogels, root mean square toughness was calculated according to the following equation:

1 $$Rq= \sqrt{\frac{1}{n} \sum_{i=1}^{n}{y}_{i}^{2}}$$

where $Rq$ is root mean square roughness, $n$ is a value of points measured, and $y$ is surface profile deviation value.

### Uniaxial tensile test with cyclic load

The mechanical properties of both raw and treated BC-hemicellulose hydrogels were evaluated using uniaxial tensile test with cyclic load. Pellicles (Fig. 1a) were cut into rectangular strips with an approximate length of 20 mm and width of 3 mm (Fig. 1b,I). The width of strips was defined as an average of three along the sample. The thickness of each specimen was measured using a BAKER IP54 digital micrometer (Baker Gauges India Private Limited, India). A tensile stage microtester (Deben Microtest, UK) with a 200N load cell was used for mechanical testing (Fig. 1b).

Samples were subjected to uniaxial tensile test with cyclic load at a strain rate of 500 µm/min, with the tensile strain increase of 500 µm per cycle. Prior to the initial loading of the first cycle, the sample was pre-loaded with a force of 0.05N to ensure fiber uncrimping. A dwelling time of 10 s was applied for stress relaxation within the sample between consecutive cycles. During the dwelling time of each consecutive cycle, sample was rehydrated by drop-deposition of 10 µL of deionized water to ensure sample hydration. Cyclic tension was performed up to the sample fracture. The tests were performed in 10 replicates at a temperature of 19 ± 1 ºC, and a relative humidity of 33 ± 2%. Schematic stress–strain curve with a graphical representation of the evaluated mechanical properties are given in Fig. 1c. Experimental stress–strain curve of raw BC, obtained by the uniaxial tensile test with cyclic load is provided in Supplementary Materials as Fig.S1.

Data from stress–strain curves were extracted using Python procedure^28^. The maximum stress and maximum strain were defined at the cycle with the highest force. Maximum modulus was defined as the maximum value of the slope of the linear part of the stress–strain curve in a single test. The total plastic strain was given as a sum of the irreversible strains in each cycle.

### Statistical analysis

Statistical analysis of experimental data was performed using *RStudio v.4.1.1* software (Posit Software, USA). Differences between monosaccharide composition, and structural and mechanical properties were analyzed using one-way analysis of variance and Tukey test at a significance level of p = 0.05.

## Results and discussion

### Dry yield of biosynthesis

**Fig. 2:**
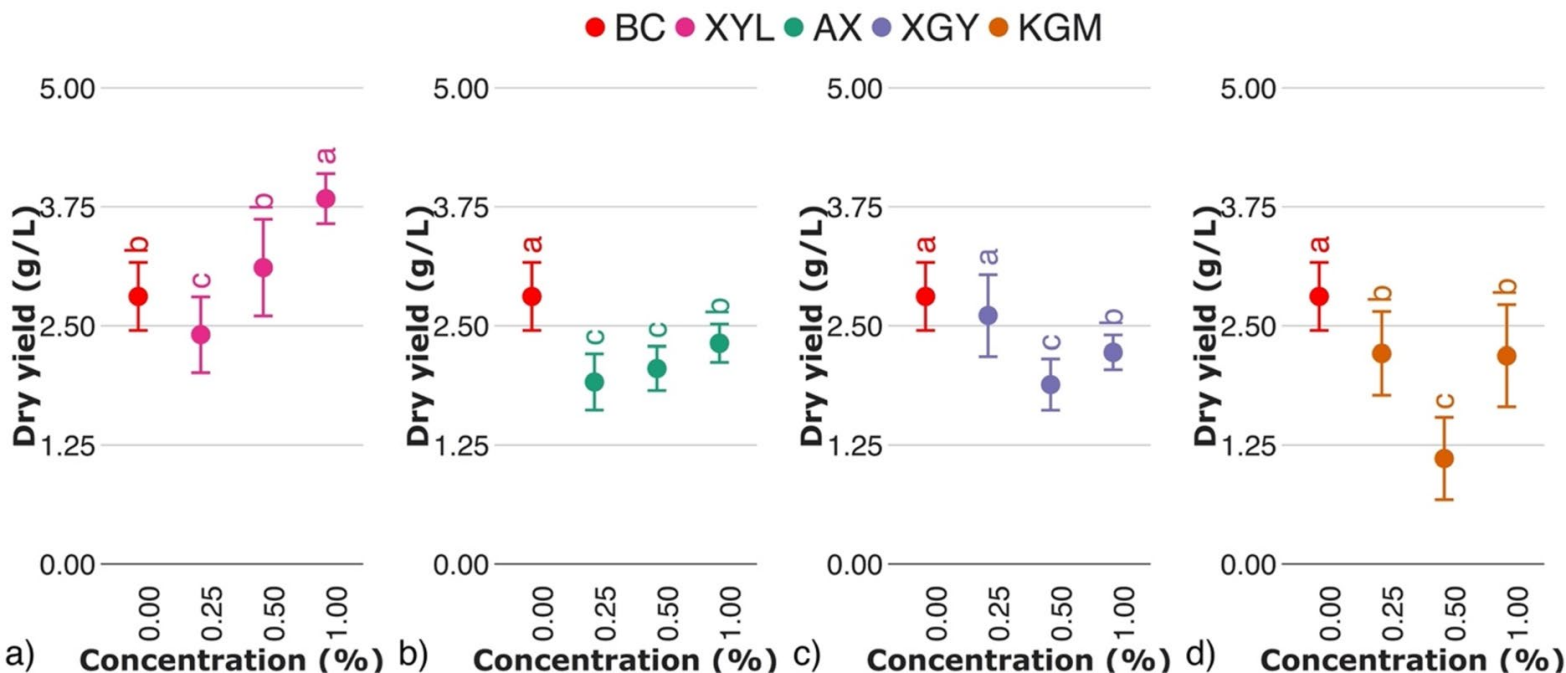
Dry yield of biosynthesis of BC hemicellulose hydrogels in relation to the presence of (**a**) xylan (XYL), (**b**) arabinoxylan (AX), (**c**) xyloglucan (XGY), and (**d**) glucomannan (KGM) in culturing medium. The control sample (BC) is marked with red colour. For the estimated parameters, the data points and bars refer to the mean values and standard deviation, respectively. Treatments with the same letter show a lack of statistically significant differences.

Produced BC-hemicellulose hydrogels (Fig. 1a) appeared as smooth layered pellicles. The dry yield of biosynthesis of pure BC was 2.8 ± 0.4 g/L (Fig. 2), being consistent with data, reported previously^16,29^.

The presence of hemicelluloses in the culturing medium resulted in a change of the dry yield of biosynthesis of respected hydrogels. An increase in dry yield of biosynthesis of BC with xylan presence was obtained compared to the pure BC (Fig. 2a). Despite the presence of glucose in the culturing medium, *K.xylinum* has also been reported to metabolise xylose^30^, being involved in bacterial growth rather than BC biosynthesis. In contrast to xylan, the addition of arabinoxylan resulted in a decrease in the dry yield of biosynthesis (Fig. 2b), showing poor ability to be utilized by *K.xylinum*^29^. Compared to unsubstituted xylan, arabinose sidechains stabilize the arabinoxylan backbone, so that the degradation of arabinoxylan backbone to xylose should be considered minor.

The presence of xyloglucan in the culturing medium resulted in a decrease in dry yield of biosynthesis (Fig. 2c), which correlates with limited water solubility of xyloglucan, restricting the mobility of bacteria and nutrients. Statistically significant higher dry yield of BC biosynthesis in xyloglucan-enriched medium was previously reported for agitated culturing^31^. Since agitated culturing conditions provide a homogeneous distribution of bacteria and nutrients within the medium, xyloglucan may act as a stabilizer of solution, while for static culturing conditions, limited solubility and possible aggregation of xyloglucan may limit BC biosynthesis. Biosynthesis of BC in the presence of glucomannan resulted in a decrease of dry yield of biosynthesis, compared to control (Fig. 2d). A similar phenomenon has been reported for other culturing media^31,32^ and is related to its limited solubility and polysaccharide over supplementation of culturing medium.

### Monosaccharide composition

**Fig. 3:**
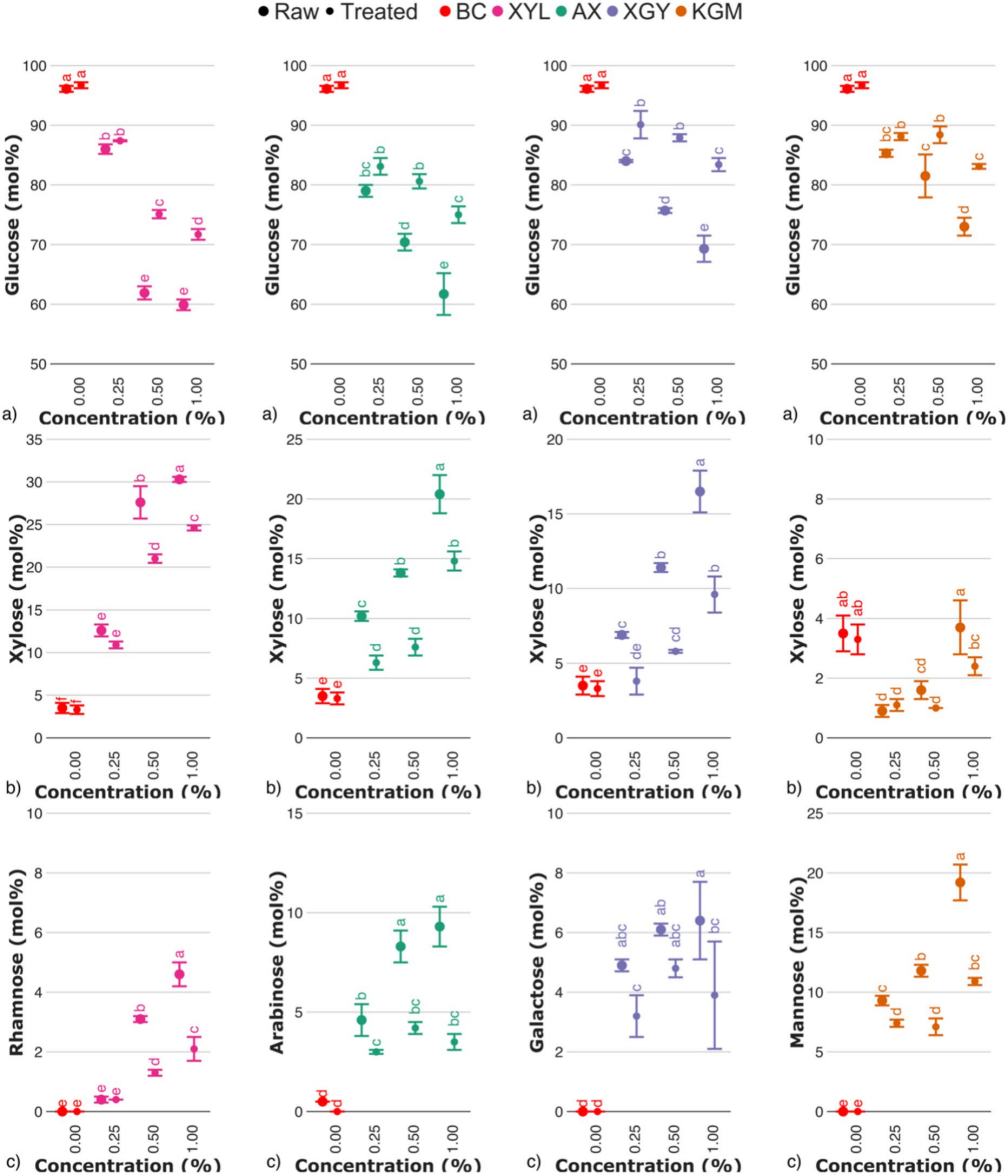
Monosaccharide composition of raw and treated BC–hemicellulose hydrogels in relation to the presence of xylan (XYL), arabinoxylan (AX), xyloglucan (XGY), and glucomannan (KGM) in culturing medium. Subsequent figures represent the content of (**a**) glucose, (**b**) xylose, and (**c**) rhamnose (for bacterial cellulose-xylan hydrogels); arabinose (for bacterial cellulose-arabinoxylan hydrogels); galactose (for bacterial cellulose-xyloglucan hydrogels) mannose (for bacterial cellulose-glucomannan hydrogels). Raw and treated samples are indicated by the bullet points of different sizes. The control sample (BC) is marked with red colour. For the estimated parameters, the data points and bars refer to the mean values and standard deviation, respectively. Treatments with the same letter show a lack of statistically significant differences. Complete data on monosaccharide composition of bacterial cellulose-hemicellulose hydrogels is provided in Fig.S2 of Supplementary Materials.

Monosaccharide composition of both raw and treated BC-hemicellulose hydrogels is provided in Fig. 3. Apart from glucose, both raw and treated BC contained trace amounts of arabinose and xylose, originating from an entrapped medium components. Raw hydrogels, cultured in the medium with specific hemicellulose additives resulted in the presence of the respected monosaccharides—glucuronic acid and xylose—for BC–xylan hydrogels; arabinose, galactose, galacturonic acid, and xylose – for BC–arabinoxylan hydrogels; galactose, galacturonic acid and xylose—for BC–xyloglucan hydrogels; mannose—for BC–glucomannan hydrogels. What is of specific interest, is that the traces of galacturonic acid were observed in BC–hemicellulose hydrogels, possible as a backbone β-D-GalpA-(1 → 2)-α-D-Xylp inclusions^33,34^. Another monosaccharide, common for pectin—rhamnose—was also reported in current samples, suggesting its appearance as a co-product of hemicellulose extraction, also being reported in reducing ends of some hemicelluloses^35–37^. Statistically significant increase of the content of hemicellulose monosaccharides in hydrogels with an increasing hemicellulose content in culturing medium allowed us to assume that hemicelluloses incorporated within respected hydrogels in a concentration–dependent manner.

For the treated BC–hemicellulose hydrogels, a statistically significant increase in glucose content was found, in addition to the reduction of the amount of hemicellulose monosaccharides. It allowed us to assume enzymolysis of hemicelluloses in respected hydrogels took place. However, enzymolysis was not complete in terms of entire hemicellulose removal, as from half to two–thirds of its initial content were still present within treated hydrogels. We suppose that incomplete enzymolysis occur since hemicelluloses are partially trapped in/within cellulose fibers, so that can only be released by cellulase treatment^9^. Thus, in this study, we assume enzyme–inaccessible hemicelluloses as an integral component of BC–hemicellulose hydrogels. Another crucial point is that despite culturing at various hemicellulose concentrations, no predominant mechanism (fiber entrapment, irreversible adsorption, etc.) of cellulose–hemicellulose binding was observed, since with an increasing hemicellulose content, both amounts of enzyme–accessible and enzyme–inaccessible hemicelluloses were increasing.

### Surface topography

**Fig. 4:**
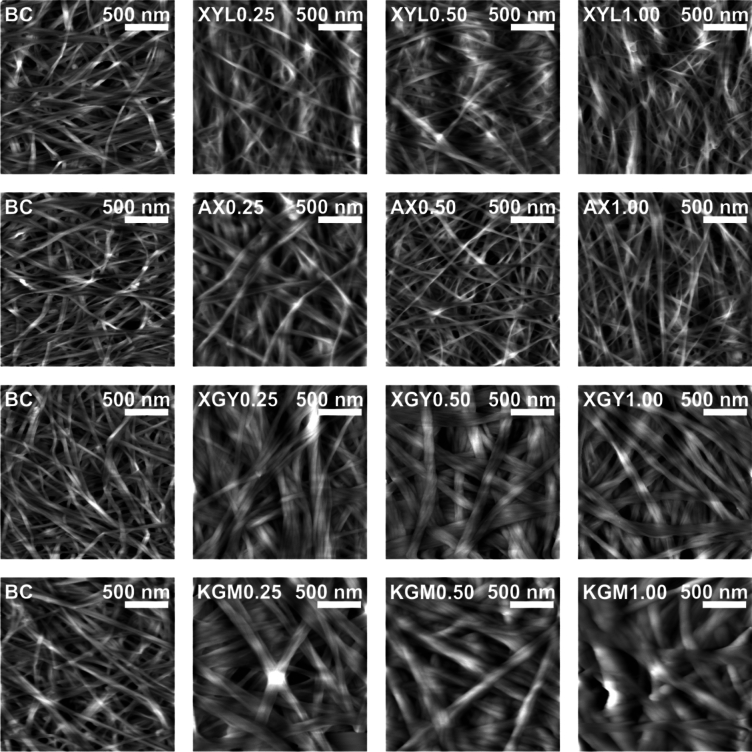
Atomic force microscopy surface topography images of raw BC hemicellulose hydrogels in a dry state. Images are divided into rows according to the type of hemicellulose additive, while column indicate the amount of hemicellulose additive (% mass) in culturing medium. For each image, abbreviation and scale are provided.

**Fig. 5:**
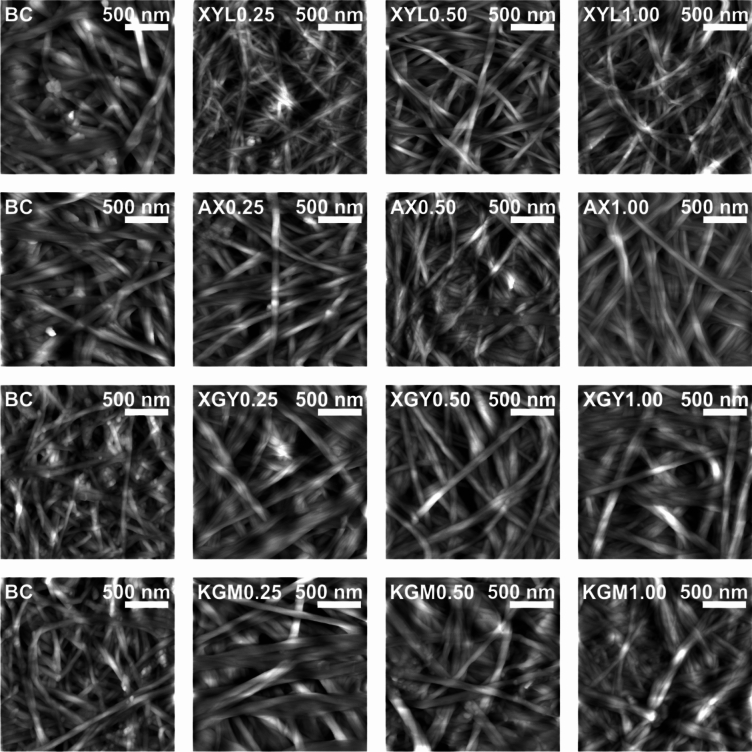
Atomic force microscopy surface topography images of treated BC hemicellulose hydrogels in a dry state. Images are divided into rows according to the type of hemicellulose additive, while column indicate the amount of hemicellulose additive (% mass) in culturing medium. For each image, abbreviation and scale are provided.

AFM surface topography revealed that both raw and treated samples appeared as a network of randomly distributed fibers (Fig. 4–5). No detectable differences in thicknesses of raw and treated BC-hemicellulose hydrogels were observed.

**Fig. 6:**
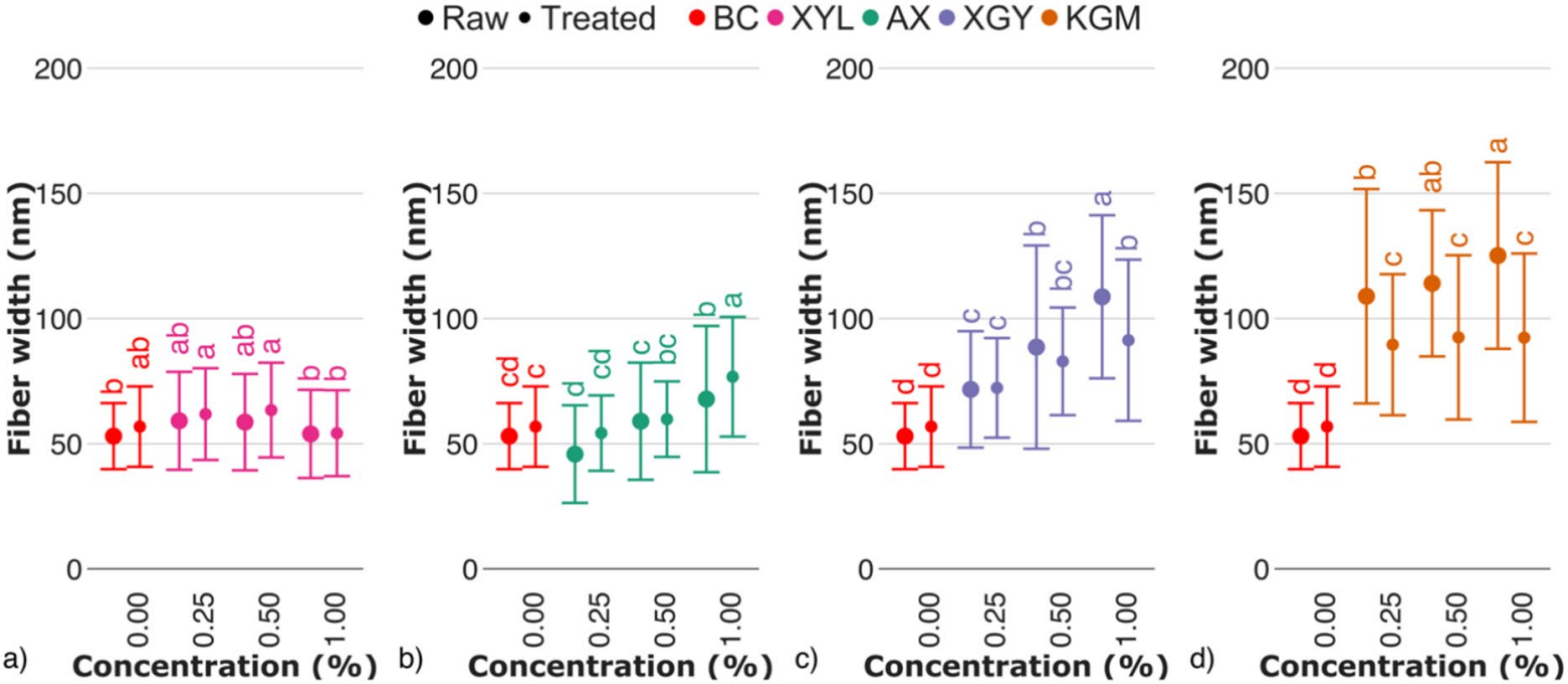
Cellulose fiber width of raw and treated BC–hemicellulose hydrogels with (**a**) xylan (XYL), (**b**) arabinoxylan (AX), (**c**) xyloglucan (XGY), and d) glucomannan (KGM) in the dry state. Raw and treated samples are indicated by the bullet points of different sizes. The control sample (BC) is marked with red color. For the estimated parameters, the data points and bars refer to mean values and standard deviation, respectively. Treatments with the same letter show a lack of statistically significant differences.

In the case of pure BC, an average fiber width of 53 ± 13 nm (Fig. 6) was observed on AFM images. In terms of length, no cellulose fiber caps were observed on either the 2 × 2 µm capturing area or the larger area tested.

With increasing xylan concentration, the fiber width of the treated samples have not changed, compared to the raw samples and pure BC (Fig. 6a). Lack of data change may be related to the xylan entrapment within cellulose fibers, or in close proximity to interfiber crosslinks, making it enzyme–inaccessible. Contrary to xylan, the addition of arabinoxylan to the culturing medium increased fiber width (Fig. 6b). We consider it to be a matter of nonspecific irreversible adsorption of arabinoxylan on the cellulose^38^, since arabinoxylan has been reported to interact with cellulose fibers by surface deposition. Fiber width has not decreased after enzymolysis, compared to raw samples. We suppose that the endo–β–1 → 4–xylanase treatment of arabinoxylan could be hindered sterically by arabinose substitutes, and as far as the removal of arabinose sidechains increases arabinoxylan adsorption affinity to cellulose^39^, the resulting change in fiber width was within the standard deviation. Another point may suggest substitution pattern define statistically significant differences in fiber width of both xylan and arabinoxylan, since it was reported to define interactions of hemicelluloses with cellulose^40^.

Fiber width increased almost twofold for the XGY1.00 sample, compared to pure BC (Fig. 6c). Fiber thickening may occur due to the adsorption of xyloglucan on the microfiber, which precedes fiber formation^41^. In addition to some entrapped xyloglucan fraction, partial xyloglucan deposition on fiber can be confirmed by the decrease in fiber width of the treated samples compared to raw.

Increasing concentration of glucomannan in the culturing medium resulted in a two– to threefold increase in fiber width in the case of the KGM1.00 sample, compared to pure BC (Fig. 6d). Heterogeneous surface structure, in which cellulose bands are hardly distinguished, was the one, specific for BC–glucomannan fiber network. It matches with the research of Szymańska–Chargot et al., who reported higher affinity of glucomannan to adsorb on apple parenchyma cellulose, compared to other hemicelluloses (Szymańska–Chargot et al.^42^). Treatment of BC–glucomannan hydrogels with dilute alkali and endo–1 → 4–β–mannanase resulted in a decrease of cellulose fiber width (Fig. 4d), reaching a plateau of about 90 ± 30 nm of fiber width. It allowed us to assume that enzymolysis enabled complete removal of enzyme–accessible glucomannan, reaching the minimum values of fiber width, determined by enzyme–inaccessible glucomannan.

**Fig. 7:**
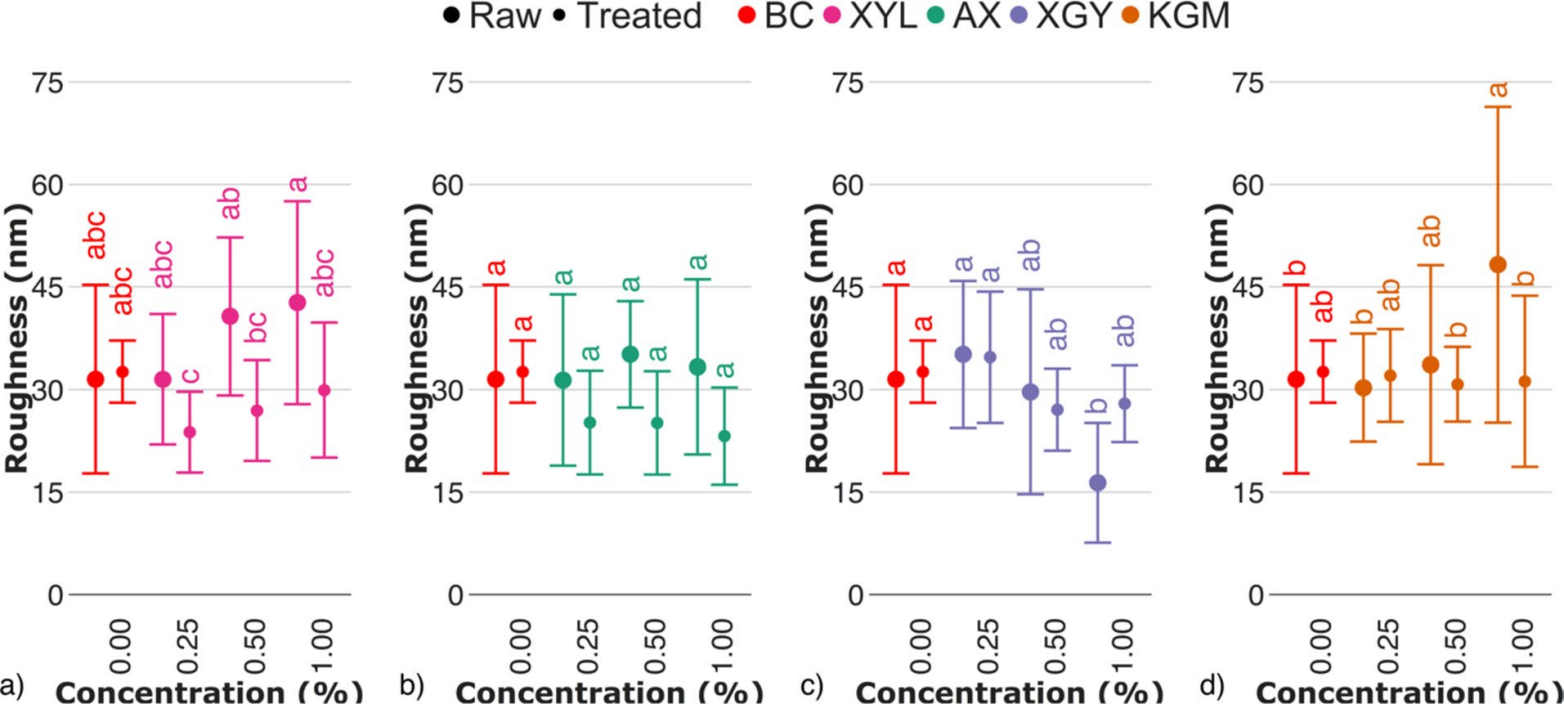
Root mean square roughness of raw and treated bacterial cellulose-hemicellulose hydrogels in relation to the presence of (**a**) xylan (XYL), (**b**) arabinoxylan (AX), (**c**) xyloglucan (XGY), and (**d**) glucomannan (KGM) in culturing medium. Raw and treated samples are indicated by the bullet points rectangles, respectively. The control sample (BC) is marked with red colour. For the estimated parameters, the data points and bars refer to the mean values and standard deviation, respectively. Treatments with the same letter show a lack of statistically significant differences.

Data on fiber width is somehow correlated with one of surface roughness, which, despite moderacy of statistical differences, was on average lower for treated samples, compared to raw (Fig. 7; Fig. S3-S4). Together with a lower data deviation for treated samples, current data may provide to a conclusion that smoother surface of oven-dried bacterial cellulose-hemicellulose hydrogels may be a matter of the removal of enzyme-accessible hemicelluloses.

### Mechanical properties of BC–hemicellulose hydrogels

**Fig. 8:**
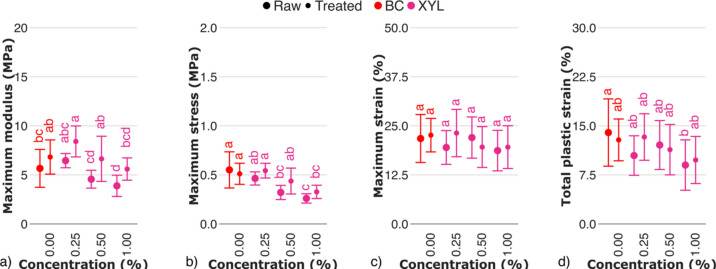
Mechanical properties of raw and treated BC–xylan hydrogels evaluated by cyclic tests: (**a**) maximum modulus, (**b**) maximum stress, (**c**) maximum strain, (**d**) total plastic strain. The control sample (BC) is marked in red. Raw and treated samples are indicated by bullet points and rectangles, respectively. For the estimated parameters, data points and bars refer to the mean and standard deviation, respectively. Treatments with the same letter show no statistically significant differences.

As the concentration of xylan in the culturing medium increased, the values of the mechanical parameters decreased statistically significant for both raw and treated samples (Fig. 8). Decreasing maximum modulus (Fig. 8a) of BC–xylan hydrogels with increasing xylan content was observed. It allowed us to suggest the mechanism, according to which xylan, entrapped within cellulose fiber, affected packaging of cellulose microfibers, and promoting the formation of an amorphous cellulose. In addition, water within hydrogel may contribute to polysaccharide chains pushing away, forcing water–induced phase separation^43^. This is consistent with our data on the decreasing maximum modulus (Fig. 8a) of BC–xylan hydrogels with increasing xylan content, suggesting that adsorbed water reduced the friction between fibers during cyclic loading and unloading. Phase separation phenomenon was also reported for other hemicelluloses studied, with the most prominent effect reported for xylan^44–46^. No statistically significant changes on maximum strain of BC-xylan hydrogels were observed (Fig. 8c), while the one of total plastic strain (Fig. 8d) were moderate.

For treated BC–xylan hydrogels, values of mechanical properties increased compared to raw samples (Fig. 8). Mechanical properties of BC–xylan hydrogels were previously shown to be determined by the conformation of the xylan chain^18^, with patterned rigid domains of the xylan chain tightly adsorbed to cellulose^47^, and non–patterned irregularly substituted domains forming flexible loops and tails between the cellulose fibers. In addition, regularity/irregularity of xylose acetylation degree may facilitate tighter binding to cellulose, defining rigidity of bacterial cellulose-xylan fiber network^48^. It allows us to suggest a mechanism, in which enzymolysis of flexible or both rigid and flexible domains of the xylan lead to direct interactions between cellulose fibers, resulting in higher moduli and stresses (Fig. 8b) in the treated samples, compared to raw. Moderate decrease in xylose content in treated BC–xylan hydrogels (Fig. 3b) somehow confirm suggested mechanism.

**Fig. 9:**
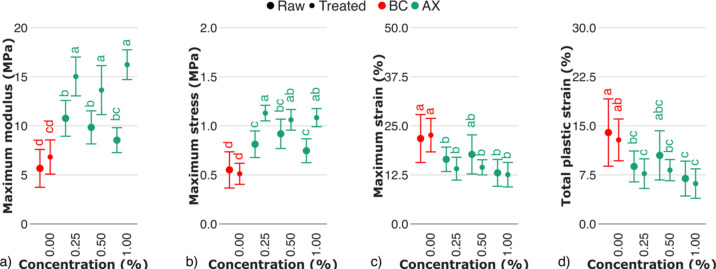
Mechanical properties of raw and treated BC–arabinoxylan hydrogels evaluated by cyclic tests: (**a**) maximum modulus, (**b**) maximum stress, (**c**) maximum strain, (**d**) total plastic strain. The control sample (BC) is marked in red. Raw and treated samples are indicated by bullet points and rectangles, respectively. For the estimated parameters, data points and bars refer to the mean and standard deviation, respectively. Treatments with the same letter show no statistically significant differences.

The mechanical properties of raw BC–arabinoxylan hydrogels showed statistically significant increase in terms of maximum modulus (Fig. 9a), and maximum stress (Fig. 9b), compared to pure BC. The dominant idea that may be considered here is that increasing maximum stress of BC–arabinoxylan hydrogels with an increasing arabinoxylan content can be attributed to its reinforcing effect^49^, with unsubstituted regions of arabinoxylan chains interacting during the plastic deformation of the sample, so that enhancing the effect of fiber realignment. In addition, arabinoxylan was not reported to incorporate into cellulose fiber as xylan^50^, affecting the packaging of cellulose chains, but mainly adsorb nonspecifically on cellulose surface^38^. The same effect was also observed in BC–arabinogalactan networks^51^, suggesting a key role of arabinose sidechains in arabinoxylan–cellulose interactions.

The values of moduli and stresses of treated BC-arabinoxylan hydrogels showed statistically significant increase, compared to raw hydrogels, contrasting with other BC-hemicellulose hydrogels by trends on data change. Low–arabinosylated arabinoxylan with a decreased degree of polymerization were shown to exhibit lower moisture uptake compared to native arabinoxylan^52^, being a matter of its higher crystallinity^39^. It is consistent with our study and suggests that increase in moduli and stresses (Fig. 9a,b), and decrease in strains (Fig. 9c,d) of BC–arabinoxylan hydrogels were defined by both the increase in polysaccharide crystallinity within the network and reduced moisture–induced plastic deformation. In addition, the removal of the arabinose sidechains increases the flexibility of the xylan chain, as well as the number of hydroxyl groups, resulting in a reinforcing effect of the flexible, low–substituted arabinoxylan.

**Fig. 10:**
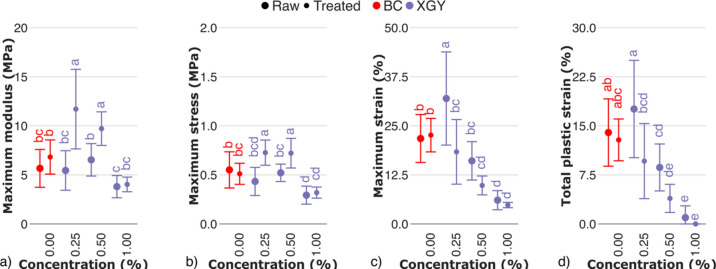
Mechanical properties of raw and treated BC–xyloglucan hydrogels evaluated by cyclic tests: a) maximum modulus, b) maximum stress, c) maximum strain, d) total plastic strain. The control sample (BC) is marked in red. Raw and treated samples are indicated by bullet points and rectangles, respectively. For the estimated parameters, data points and bars refer to the mean and standard deviation, respectively. Treatments with the same letter show no statistically significant differences.

Contrary to both xylan and arabinoxylan, the presence of xyloglucan and glucomannan in culturing medium decreased the mechanical properties of the respective hydrogels. With increasing xyloglucan content, the values of maximum modulus, maximum stress, maximum strain, and total plastic strain decreased with a treatment–dependent trend (Fig. 10). Xyloglucan was observed to deposit on cellulose surface during fiber extrusion by bacteria^53^. With a sequential increase of xyloglucan content in culturing medium, its deposition within the cellulose fibers also increases, reducing a number of stiff cellulose–cellulose junctions. In that case, we consider mechanical properties of BC–xyloglucan hydrogels are predominantly determined by weaker xyloglucan–cellulose interactions, occurring both on surface of cellulose fiber and between cellulose microfibers forming fiber^42^, which conceptually similar to PCW biomechanical hotspots^9^ rather than stronger cellulose–cellulose interactions. Such a xyloglucan fraction is enzyme–inaccessible, acts as a mediate of both microfibers and fibers, and determines the trends of data change. At the same time, the presence (adsorption, adhesion, entrapment) or absence (solubilisation, enzymolysis) of enzyme–accessible xyloglucan determines data fluctuation.

Suggested role of enzyme–accessible xyloglucan correlates with a statistically significant increase of moduli (Fig. 10a), and stresses (Fig. 10b) of treated samples compared to raw. As xyloglucan content in hydrogel increases, the maximum stress is determined more by enzyme–inaccessible xyloglucan–mediated interfiber interactions, so that the effect of xyloglucan enzymolysis is limited.

In contrast to the moduli and stresses, the maximum strain (Fig. 10c) was higher for raw samples, compared to treated. The total plastic strain of BC-xyloglucan hydrogels decreased (Fig. 10d) with an increasing xyloglucan content, being governed by a reduction of interfiber friction by an amorphous xyloglucan.

**Fig. 11:**
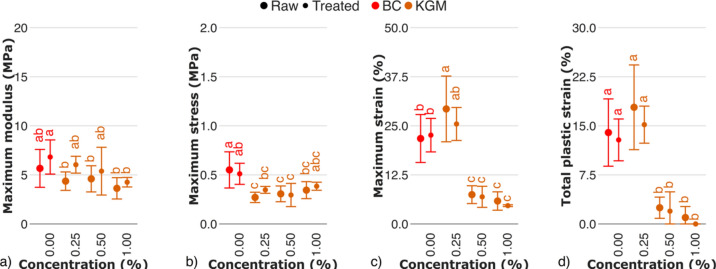
Mechanical properties of raw and treated BC–glucomannan hydrogels evaluated by cyclic tests: (**a**) maximum modulus, (**b**) maximum stress, (**c**) maximum strain, (**d**) total plastic strain. The control sample (BC) is marked in red. Raw and treated samples are indicated by bullet points and rectangles, respectively. For the estimated parameters, data points and bars refer to the mean and standard deviation, respectively. Treatments with the same letter show no statistically significant differences.

The decrease of the mechanical properties of BC–glucomannan hydrogels was more prominent, compared to xyloglucan (Fig. 11). An increase in glucomannan content resulted in a moderate decrease in moduli (Fig. 11a), and stresses (Fig. 11b). However, the change in maximum strain of the BC–glucomannan hydrogels was steep, decreasing from 22.6 ± 4.3% to 4.7 ± 0.2% for BC and KGM1.00, respectively (Fig. 11c). At low glucomannan content, increase in mechanical properties is hypothesized to occur due to co–crystallization of low amounts of semi–crystalline glucomannan with BC^54^, while with an increasing glucomannan content, decrease in mechanical properties is determined by an increasing glucomannan deposition on cellulose^55^. Due to the structural similarity of cellulose and glucomannan, it not only exhibits good binding affinity to cellulose, but also prevents direct assembly of microfibers and/or fibers^54^. The *in situ* interaction between glucomannan and bacteria–extruded microfibers can lead to the disruption of cellulose chain packaging^56^, resulting in a formation of fragile fibers and fiber network, compared to pure BC.

In terms of mechanical properties, enzymolysis of the BC–glucomannan hydrogels resulted in moderate data change. The higher maximum strain for raw samples (Fig. 11c), compared to treated, might indicate the role of glucomannan in resisting load at high strain, with a less pronounced effect compared to BC–xyloglucan hydrogels. Despite the decrease in fiber width for the treated BC–glucomannan hydrogels, the values were still higher compared to the control, suggesting that a part of glucomannan is enzyme–inaccessible. Therefore, the thicker fibers of raw samples resulted in limited entanglement and interfiber interactions within the network, leading to a lower maximum modulus, compared to the treated samples.

Similar to other hemicelluloses studied, glucomannan decreased the total plastic strain of BC–glucomannan hydrogels (Fig. 11d). At low glucomannan content in culturing medium, its deposition on cellulose is lower, compared to water, so irreversible deformations are likely defined by a weakened hydrogen bonding network of water–mediated cellulose–cellulose and cellulose–glucomannan slip–stick interactions^57^. With increasing content, glucomannan predominantly deposits on cellulose, forming a strong hydrogen bonding network. The data obtained are consistent with the predominantly reversible deformation of cellulose–glucomannan hydrogels to cyclic compression, observed previously^18^. Moreover, observations on fiber width allowed to hypothesize that the decrease of irreversible deformation of BC–glucomannan hydrogels was governed by the limited formation of loops of thick fibers, so that interfiber friction define irreversibility of deformation was governed by interfiber friction.

## Conclusion

In current study we have investigated the interactions between bacterial cellulose (BC) and four hemicelluloses—arabinoxylan, xylan, xyloglucan, and glucomannan—in hydrogels. By examining the effects of these hemicelluloses on the structure, mechanical properties, and biosynthesis of BC-hemicellulose hydrogels, we were able to identify how the distinct structures and compositions of each hemicellulose influence these interactions.

Low-molecular-weight xylan and arabinoxylan had minimal impact on BC biosynthesis. In contrast, high-molecular-weight xyloglucan and glucomannan altered hydrogel formation due to increased medium viscosity and limited nutrient mobility. Removal of enzyme-accessible hemicelluloses, particularly xylan and xyloglucan, generally improved the mechanical properties of the hydrogels by enhancing cellulose-cellulose interactions.

The study also found that increasing xyloglucan content weakened the mechanical properties of BC–xyloglucan hydrogels due to weaker xyloglucan–cellulose interactions, while higher arabinoxylan content led to stronger hydrogels due to reinforced cellulose fibers. Glucomannan's impact on mechanical properties was less pronounced but similar to xyloglucan, with fiber network formation being altered by glucomannan deposition on and within the cellulose fibers.

These findings demonstrate the structure-dependent mechanisms of cellulose–hemicellulose interactions and suggest specific structural roles for each hemicellulose in determining the properties of BC–hemicellulose hydrogels. The results have potential applications in developing composite biomaterials for scientific and commercial use.

## Supplementary Information

Supplementary Figures.

## Data Availability

Data will be made available on request. For data request, please contact v.chibrikov@ipan.lublin.pl.
