# Supplementary material for: The effect of hemicelluloses on biosynthesis, structure and mechanical performance of bacterial cellulose-hemicellulose hydrogels
Source: Sci Rep. 2024 Sep 17;14:21671. doi: 10.1038/s41598-024-72513-w (PMC11408599; doi:10.1038/s41598-024-72513-w)
Supplement: Supplementary file 1 — Supplementary Figures. [file 41598_2024_72513_MOESM1_ESM.docx]

**The effect of hemicelluloses on biosynthesis, structure and mechanical performance of bacterial cellulose-hemicellulose hydrogels**

Vadym Chibrikov _a_, Piotr Mariusz Pieczywek _a_, Justyna Cybulska _a_, Artur Zdunek _a_*

_a_ *Institute of Agrophysics, Polish Academy of Sciences, Doświadczalna 4  Str., Lublin 20*–*290, Poland*

*corresponding author: Artur Zdunek, Institute of Agrophysics, Polish Academy of Sciences, Doświadczalna 4 Str., 20–290 Lublin, Poland, tel. +48817445061 int. 103, e–mail: [a.zdunek@ipan.lublin.pl](mailto:a.zdunek@ipan.lublin.pl)

Author details

Vadym Chibrikov e–mail: [v.chibrikov@ipan.lublin.pl](mailto:v.chibrikov@ipan.lublin.pl)

ORCID: https://orcid.org/0000-0002-2007-3800

Piotr Mariusz Pieczywek e–mail: [p.pieczywek@ipan.lublin.pl](mailto:p.pieczywek@ipan.lublin.pl)

ORCID: https://orcid.org/0000-0002-3986-3802

Justyna Cybulska e–mail: [j.cybulska@ipan.lublin.pl](mailto:j.cybulska@ipan.lublin.pl)

ORCID: https://orcid.org/0000-0003-3323-4535

Artur Zdunek e–mail: [a.zdunek@ipan.lublin.pl](mailto:a.zdunek@ipan.lublin.pl)

ORCID: https://orcid.org/0000-0001-9395-1486


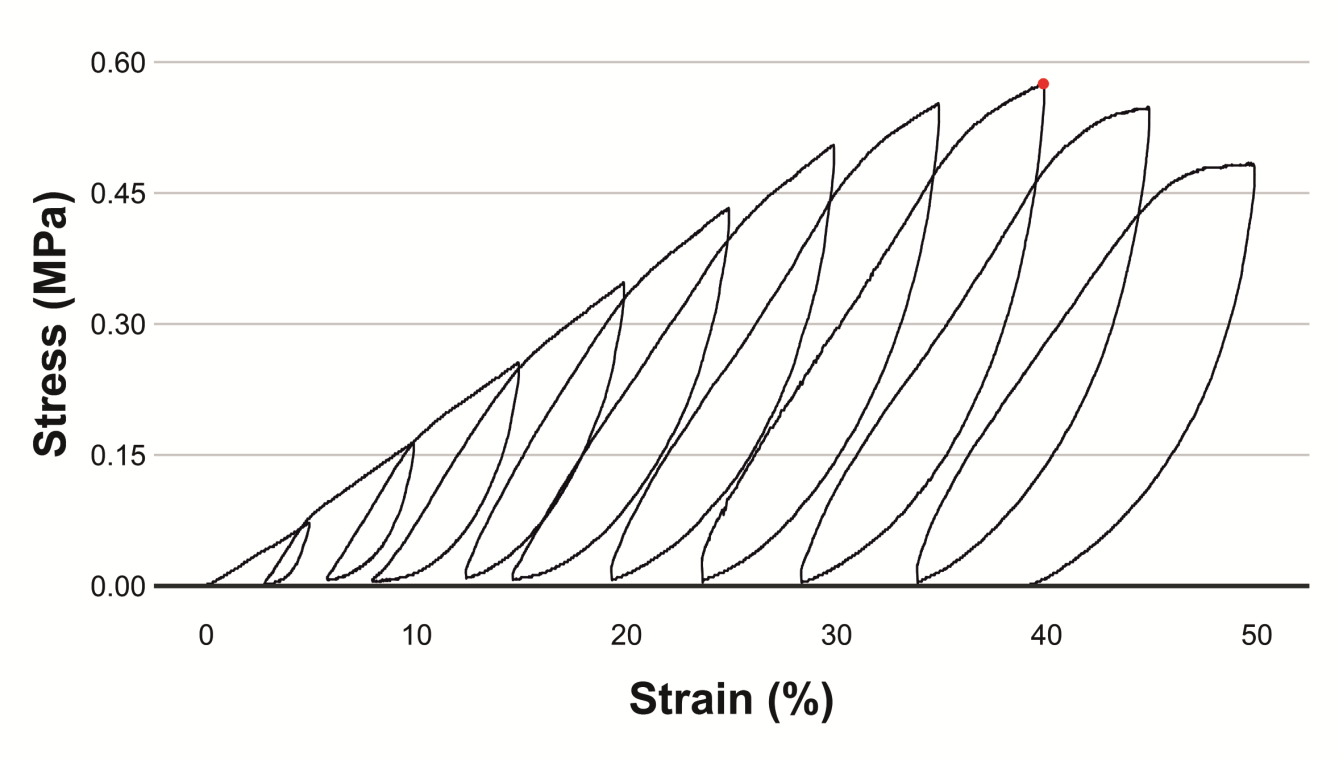


**Fig.S1.** Experimental stress‑strain curve of raw BC, obtained by uniaxial tensile test with cyclic load. Original curves were captured as elongation‑force one, converted to stress‑strain curves, following by data smooth with convolution filter. The single deformation cycle was divided into a loading (up to a maximum stress reached within the applied strain increment), and an unloading (up to zero stress reached) stages. Deformation cycles were repeated with the constant tensile strain increments up to decrease of maximum force within two consecutive cycles. The maximum stress and maximum strain were defined as that, reached at the cycle with the highest stress value (marked with red point).


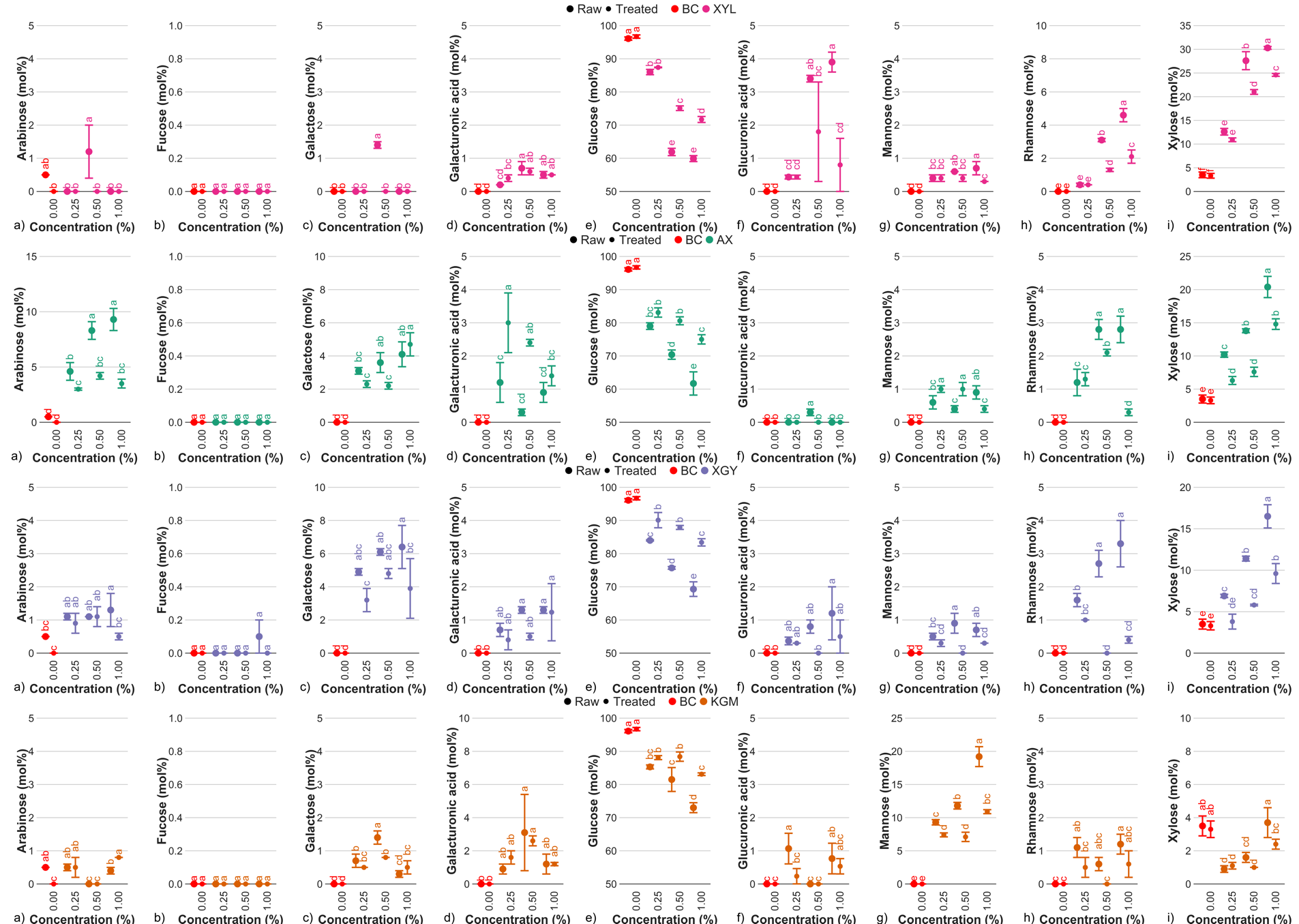


**Fig.S2.**Monosaccharide composition of raw and treated BC–hemicellulose hydrogels in relation to the presence of xylan (XYL), arabinoxylan (AX), xyloglucan (XGY), and glucomannan (KGM) in culturing medium. Subsequent figures represent the content of a) arabinose, b) fucose, c) galactose, d) galacturonic acid, e) glucose, f) glucuronic acid, g) mannose, h) rhamnose, and i) xylose in samples studied. Raw and treated samples are indicated by the bullet points rectangles, respectively. The control sample (BC) is marked with red colour. For the estimated parameters, the data points and bars refer to the mean values and standard deviation, respectively. Treatments with the same letter show a lack of statistically significant differences.


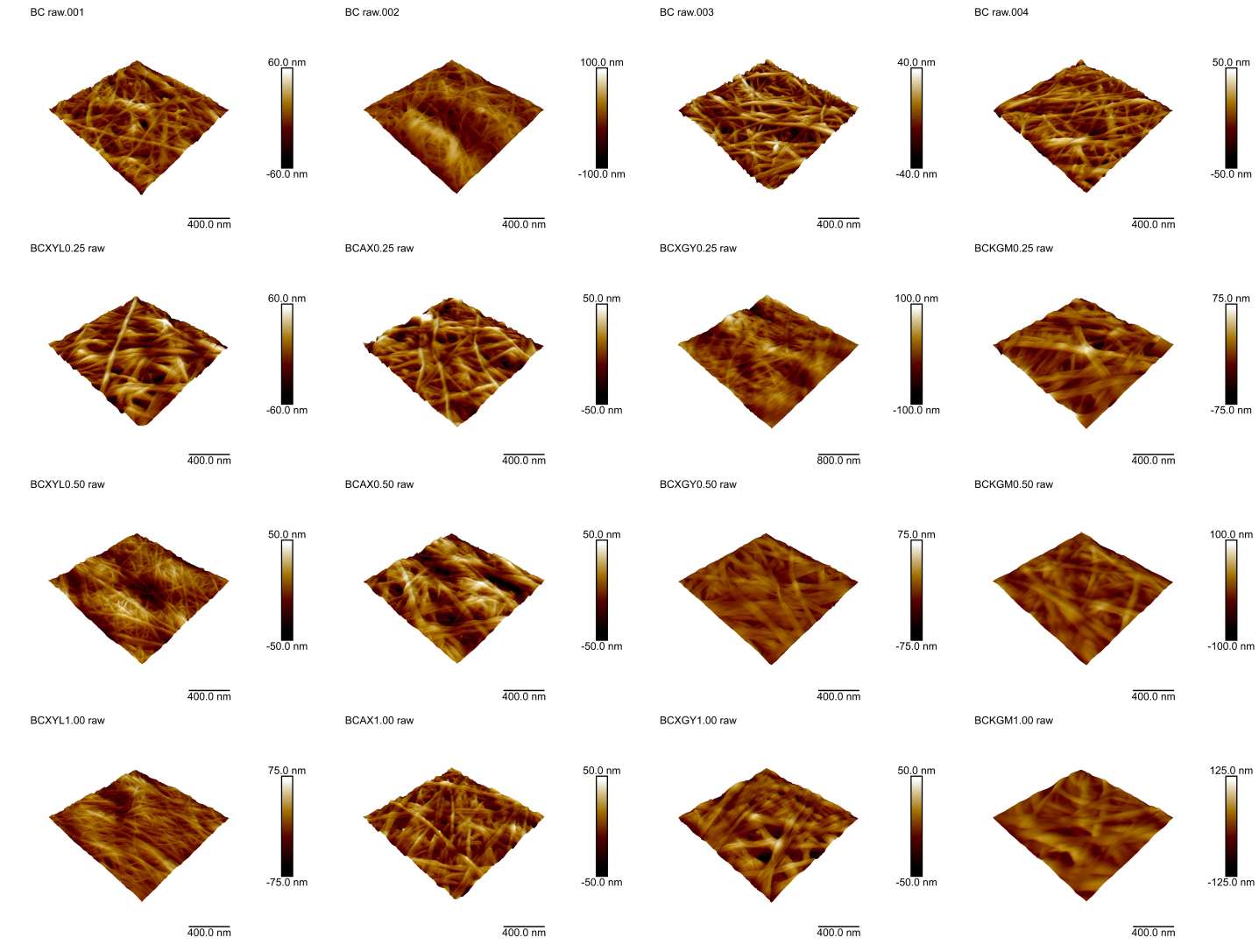


**Fig.S3.** Three-dimensional surface representation of raw bacterial cellulose-hemicellulose hydrogels in relation to the presence of xylan, arabinoxylan, xyloglucan, and glucomannan in culturing medium. Sample naming convention includes bacterial cellulose (BC), presence of hemicelluloses (XYL for xylan, AX for arabinoxylan, XGY for xyloglucan, and KGM for glucomannan), and concentration of hemicelluloses in culturing medium (% w/v). Colorbar of each image encodes values in Z axis.


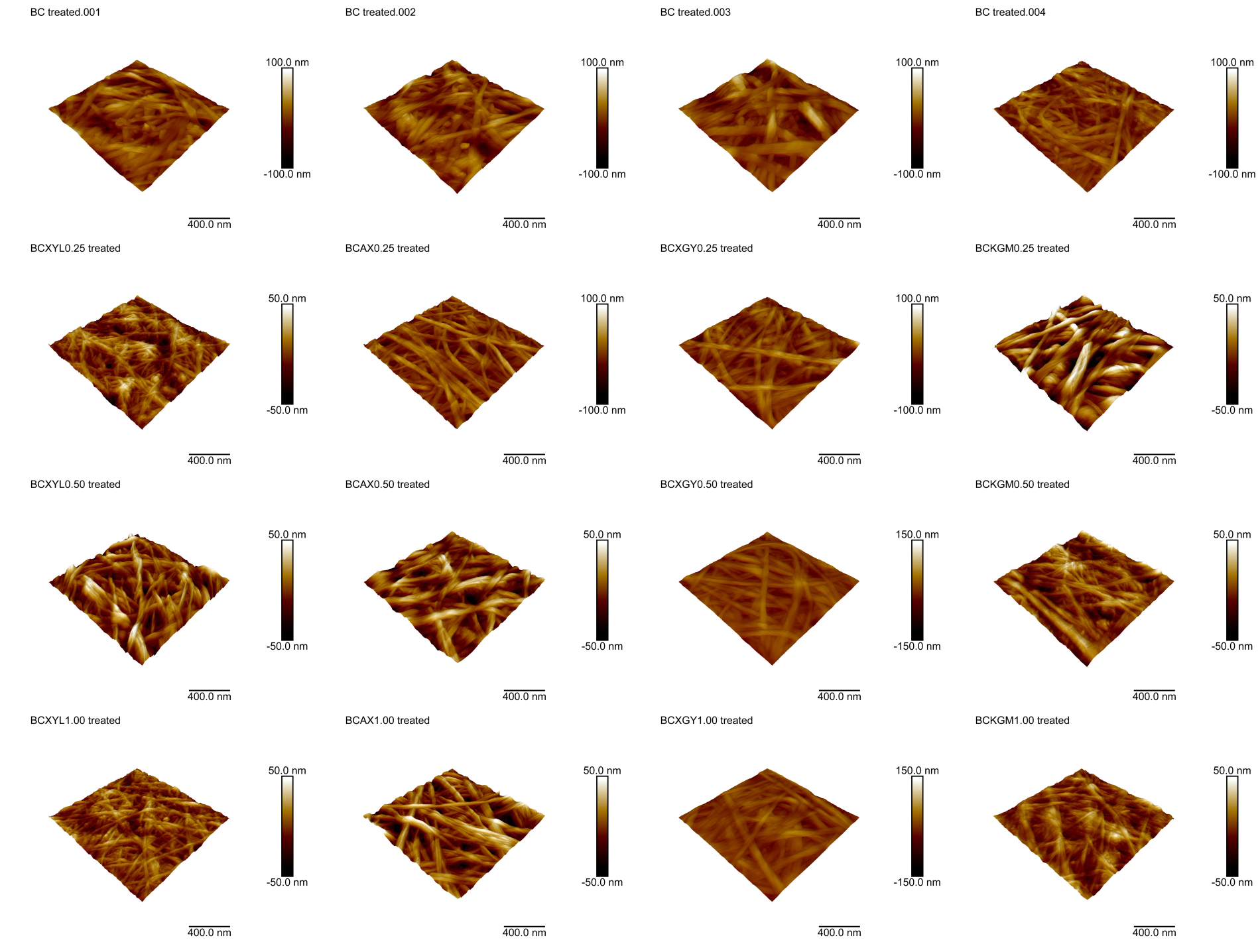


**Fig.S4.** Three-dimensional surface representation of treated bacterial cellulose-hemicellulose hydrogels in relation to the presence of xylan, arabinoxylan, xyloglucan, and glucomannan in culturing medium. Sample naming convention includes bacterial cellulose (BC), presence of hemicelluloses (XYL for xylan, AX for arabinoxylan, XGY for xyloglucan, and KGM for glucomannan), and concentration of hemicelluloses in culturing medium (% w/v). Colorbar of each image encodes values in Z axis.
